# Hepatitis C Virus (HCV) Infection: Pathogenesis, Oral Manifestations, and the Role of Direct-Acting Antiviral Therapy: A Narrative Review

**DOI:** 10.3390/jcm13144012

**Published:** 2024-07-09

**Authors:** Dario Di Stasio, Agostino Guida, Antonio Romano, Massimo Petruzzi, Aldo Marrone, Fausto Fiori, Alberta Lucchese

**Affiliations:** 1 Multidisciplinary Department of Medical-Surgical and Dental Specialties, University of Campania “Luigi Vanvitelli”, 81100 Naples, Italyaldo.marrone@unicampania.it (A.M.); alberta.lucchese@unicampania.it (A.L.); 2 U.O.C. Odontostomatologia, A.O.R.N. “A. Cardarelli”, 95123 Naples, Italy; 3 Section of Dentistry, Interdisciplinary Department of Medicine (DIM), University “Aldo Moro” of Bari, Clinica Odontoiatrica del Policlinico di Bari, Piazza Giulio Cesare 11, 70124 Bari, Italy

**Keywords:** hepatitis C, extra-hepatic manifestations, oral mucosa, oral cavity

## Abstract

Hepatitis C virus (HCV) infection is a global health concern with significant systemic implications, including a range of oral manifestations. This review aims to provide a comprehensive overview of the oral and dental pathologies related to HCV, the etiopathogenetic mechanisms linking such conditions to HCV and the impact of direct-acting antiviral (DAA) therapy. Common oral manifestations of HCV include oral lichen planus (OLP), periodontal disease, and xerostomia. The pathogenesis of these conditions involves both direct viral effects on oral tissues and indirect effects related to the immune response to HCV. Our literature analysis, using PubMed, Scopus, Web of Science, and Google Scholar, suggests that both the HCV infection and the immune response to HCV contribute to the increased prevalence of these oral diseases. The introduction of DAA therapy represents a significant advancement in HCV treatment, but its effects on oral manifestations, particularly OLP, are still under evaluation. Although a possible mechanism linking HCV to OSCC is yet to be determined, existing evidence encourages further investigation in this sense. Our findings highlight the need for established protocols for managing the oral health of patients with HCV, aiming to improve outcomes and quality of life.

## 1. Introduction

Hepatitis C virus (HCV) infection is a critical public health issue, affecting millions worldwide. It is primarily known for its impact on liver function, but its systemic nature means that its effects can be far-reaching, including the oral cavity [1]. 

The global burden of HCV is substantial, with varying prevalence rates across different regions. The introduction of direct-acting antivirals (DAAs) has revolutionized the treatment landscape [2]. However, the oral implications of HCV, particularly in the context of oral lichen planus (OLP), xerostomia, and Sjögren’s syndrome-like manifestations, periodontal diseases, and head and neck squamous cell carcinoma (HNSCC) require further exploration.

This narrative review seeks to elucidate the various oral manifestations of HCV, with a special focus on OLP [3]. Drawing from a range of sources, including a detailed cohort study on the impact of DAAs on HCV-related OLP, this review aims to provide a comprehensive overview of the current state of knowledge in this field [4].

### 1.1. Pathophysiology

HCV is a small, enveloped RNA virus from the Flaviviridae family [5]. It primarily targets hepatocytes, leading to chronic inflammation, fibrosis, and potentially cirrhosis or hepatocellular carcinoma [6]. Beyond the liver, HCV affects various systems, including the oral cavity [7]. The virus’s interaction with the immune system is critical for its persistence and the development of extrahepatic manifestations, such as OLP [8].

#### 1.1.1. Viral Entry and Replication

HCV entry into liver cells involves interactions between viral envelope proteins (E1 and E2) and host cell receptors like CD81, SR-B1, CLDN1, and OCLN, facilitating endocytosis [9]. Once inside, the viral RNA genome is released and translated into a polyprotein, which is cleaved into structural and non-structural proteins. The non-structural proteins form a replicase complex that synthesizes new viral RNA, highlighting the virus’s dependence on the host’s lipid metabolism and VLDL synthesis pathway [10,11,12].

#### 1.1.2. Immune Response and Liver Damage

HCV infection triggers the innate immune system via pattern recognition receptors, leading to the production of type I and III interferons (IFNs). These IFNs induce an antiviral state in hepatocytes and activate immune cells like NK cells and macrophages [13]. The adaptive immune response involves B-cells producing specific antibodies and T-cells targeting infected hepatocytes [14]. Chronic HCV infection often results in an insufficient T-cell response, leading to persistent inflammation, hepatocyte injury, and the activation of hepatic stellate cells, promoting fibrosis [15]. HCV evades the immune response through rapid mutation, the suppression of IFN pathways, and the induction of immune cell exhaustion, complicating viral clearance [16,17].

#### 1.1.3. Chronic Infection and Fibrosis

Chronic HCV infection maintains liver inflammation, activating hepatic stellate cells (HSCs) to produce excessive extracellular matrix, leading to fibrosis and cirrhosis [18,19]. This process is mediated by cytokines like TGF-β and involves interactions between HSCs, immune cells, and liver epithelial cells. Chronic inflammation and oxidative stress perpetuate fibrosis, disrupting liver architecture and function [20,21]. Advanced fibrosis results in cirrhosis, impairing liver function and causing complications such as portal hypertension and liver failure [22].

#### 1.1.4. Extrahepatic Manifestations

HCV’s systemic effects include immune complex formation, cryoglobulinemia, and direct viral effects on various tissues. These mechanisms lead to conditions like mixed cryoglobulinemia syndrome, neuropsychiatric disorders, thyroid dysfunction, renal disease, pulmonary conditions, dermatological manifestations, and ocular diseases [23,24,25,26]. Cryoglobulinemia, involving immunoglobulins that precipitate in the cold, can cause organ damage through hyperviscosity syndrome or immune-mediated mechanisms [27]. Research shows HCV RNA in B-cells, indicating viral replication within these cells. This interaction with the immune system, particularly through the CD81 receptor on B-cells, plays a significant role in HCV pathogenesis and the development of cryoglobulinemia, which is closely linked to lymphoproliferative disorders and an increased risk of non-Hodgkin’s lymphoma [28,29].

## 2. Oral Manifestations

OLP, xerostomia, and periodontal disease have been reported as oral manifestations in patients with HCV infection. These associations are thought to be mediated through both direct viral effects on oral tissues and indirect effects related to the immune response to HCV [30]. 

The relationship between HCV and these oral manifestations is underpinned by both direct viral effects and the virus’s impact on the immune system, leading to inflammatory and autoimmune responses within the oral cavity [31]. The advent of direct-acting antivirals (DAAs) has begun to change the landscape, with emerging evidence suggesting improvements in oral health outcomes among treated individuals [32].

### 2.1. OLP

OLP is a chronic inflammatory condition of the mucous membranes, often presenting with white, lacy patches, or red, swollen tissues [33]. A significant body of research has explored the association between OLP and HCV infection, with varying prevalence rates reported globally [1]. This association has been a subject of significant research interest, leading to new insights into pathogenesis and management [34]. The global prevalence of HCV in patients with OLP is reported to be variable, with a recent meta-analysis indicating that OLP patients have a four-fold higher frequency of HCV compared to controls. This prevalence exhibits geographical variability. When analyzing the prevalence of OLP in HCV-infected patients across different geographic regions, the African and Southeast Asian regions showed the highest odds ratios of 8.57 and 7.73, respectively. In contrast, studies from the European region did not demonstrate a significant association (OR 2.08 [0.95–4.52]). On a country level, Iraq and Egypt exhibited nearly ten-fold increases in risk, highlighting significant regional variations [35]. This study also suggested that geographical variability could be due to immunogenetic influences, such as different HCV genotypes and human leukocyte antigens (HLA). HCV genotypes 1a and 1b are most common in LP patients with HCV, varying by region. For example, in India, 70% of LP patients had genotype 1b compared to 34.1% in donors [35]. Genotype 3 is predominant in the UK, and genotype 4 is prevalent in Egypt.

Moreover, the association is strong in the Eastern Mediterranean (OR 5.51) but not in Europe (OR 1.47). Some studies detected HCV RNA in skin and oral mucosa, suggesting possible epithelial tropism. The lymphotropic nature of HCV, causing B-lymphocyte expansion and autoimmune responses, might contribute to LP. Increased oxidative stress in HCV patients also plays a role [35].

The pathogenesis of OLP in HCV-infected patients is complex and not fully understood. It is suggested that HCV replicates in the oral mucosa, leading to a localized immune response. The presence of HCV-specific T lymphocytes in the oral mucosa of OLP patients implies the potential involvement of HCV in its pathogenesis. There is also evidence of circulating antibodies to epithelial antigens in some patients with HCV-associated OLP, although their precise role in disease development remains unclear [8]. A recent review suggested that the pathogenesis of OLP involves a complex interplay of immune responses, with dysbiosis in the oral microbial community and altered immune pathways potentially playing a significant role. In particular, pathways involved in defense against bacterial infection and inflammatory responses are activated in OLP-associated microbiomes [36]. Molecular studies have found HCV RNA in oral lichen tissue, indicating sporadic HCV replication in these lesions. However, the exact mechanism by which HCV contributes to OLP remains to be fully elucidated [37]. In fact, the association between HCV and oral lichen planus (OLP) may be due to the virus’s ability to replicate in the skin and oral mucosa. While HCV replication is predominantly observed in hepatocytes, some studies have detected viral RNA in the skin and oral mucosa of patients with chronic hepatitis C, regardless of the presence of lichen planus (LP) lesions. However, other studies have failed to find HCV RNA in these tissues, indicating that evidence supporting the epithelial tropism of HCV is currently insufficient [38]. 

The cytokine profile in HCV-associated OLP suggests an immune response characterized by excessive production of Th1 cytokines following an ineffective antiviral immune response [39]. 

Various studies have examined the roles of different cytokines in this context:

- Th1 cytokines and immune response: El-Howati et al. emphasize the role of CD8+ cytotoxic and CD4+ Th1 polarized T-cells in OLP, noting the involvement of other Th subsets such as Th9, Th17, and Tregs in the disease’s pathogenesis. They suggest that both direct effects of HCV on the immune system and broader dysregulation contribute to OLP [40]. Studies have consistently shown an increased production of Th1 cytokines, including TNF-alpha, which indicates a strong Th1-mediated immune response [39].
- Role of CD8+ cytotoxic T-cells: CD8+ T-cells are crucial for targeting HCV-infected cells. However, the high mutation rate of HCV often leads to immune escape, resulting in chronic infection. This mechanism is well-documented and highlights the challenges in clearing the virus [41].
- Helper T-cells and sustained Th1 response: Helper T-cells assist in maintaining the function of CD8+ T-cells and in cytokine production. In chronic HCV infection, this leads to a sustained Th1 response, which can become dysregulated over time. This prolonged response contributes to the pathology observed in OLP [42].
- Th9, Th17, and regulatory T-cells (Tregs): Th9 and Th17 cells are associated with inflammation and tissue damage, which are characteristic of chronic infections. Studies have shown that these cells contribute to the immunopathogenesis of OLP [42]. Tregs help maintain immune tolerance and prevent autoimmune responses. Their altered function during HCV infection is a significant factor in the disease progression [42].
- Salivary cytokine profiles in OLP patients: Research on salivary cytokine profiles in OLP patients has found higher concentrations of IL-2, IL-23, and TGF-β, suggesting these cytokines play a role in OLP pathogenesis [43]. Askoura et al. reported elevated levels of IL-33, IL-17, and IL-25 in HCV patients, indicating their involvement in inflammation and the progression of fibrosis [44].
- Cytokines and disease prognosis: Zhu et al. highlighted a set of cytokines/chemokines correlated with disease prognosis in chronic liver disease, which is relevant for understanding OLP associated with HCV [45]. Vičić et al. reviewed the immunopathogenesis of lichen planus, emphasizing the complex interplay of immune cells and inflammatory pathways in HCV-associated OLP [46].
- Impact of HCV eradication on cytokine profiles: Radmanić et al. evaluated the impact of HCV eradication on cytokine and growth factor profiles, providing insights into potential changes in the cytokine environment in OLP following HCV treatment [47].

**Figure 1:**
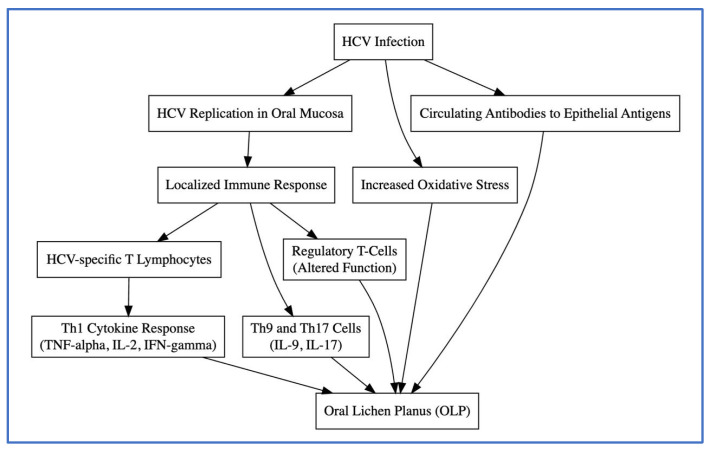
Aetiopathogenic hypotheses of the relationship between HCV infection and OLP.

In summary, as shown in Figure 1, while different studies focus on various cytokines and immune responses, the overarching theme is the multifaceted immune dysregulation driven by both antiviral and inflammatory responses in HCV-associated OLP. 

The variable effect of antiviral therapy, including interferon-alpha (IFN-alpha) with or without ribavirin on OLP has been noted, with some patients experiencing an improvement in OLP lesions following HCV treatment [48]. This suggested a potential direct role of HCV in the pathogenesis of OLP, although this is not a consistent finding across all patients. In fact, some studies reported that OLP occurred, exacerbated, and persisted during IFN treatment for hepatitis C, even when serum HCV RNA became negative. The improvement of the lesions of OLP after the discontinuation of therapy suggests a role of IFN in inducing or worsening these lesions in some patients [45,49]. Moreover, the combination of interferon (IFN) and ribavirin (RBV) achieve sustained virological response (SVR) rates of only 40–50% in patients with genotype 1 and is associated with significant side effects (gastrointestinal, hematological, and psychiatric). The currently developed IFN-free, direct-acting antivirals (DAAs) used to treat HCV infection have low side effect profiles and high efficacy (SVR > 90) [50]. Some recent studies have reinforced the notion that modern DAAs improve OLP clinical outcomes in HCV-infected patients. This evidence supported the hypothesis that successful antiviral therapy against HCV led to improvements in OLP symptoms [4,51]. A study conducted by our research group emphasized that OLP-HCV patients displayed more severe clinical symptoms at baseline and greater erosive areas compared to non-HCV-OLP patients. Post-DAA treatment, the clinical progression in OLP-HCV patients mirrored that of the non-HCV-OLP group, reinforcing the detrimental impact of HCV on OLP. Interestingly, ulcerative lesions increased temporarily after DAA treatment but improved significantly thereafter. Following HCV eradication, some patients achieved complete mucosal healing, underscoring the potential of DAAs to improve both hepatic and extra-hepatic manifestations of HCV. These findings suggest that HCV acts as a pathogenic cofactor in OLP, advocating for routine HCV testing in severe OLP cases, especially in regions with high HCV prevalence like Italy. Further research with larger samples is needed to validate these results and explore the underlying mechanisms [4]. 

### 2.2. Xerostomia and Sjögren’s Syndrome-like Manifestations

The molecular mechanisms underlying xerostomia associated with HCV infection involve a multifactorial process. Lymphocytic infiltrates in the salivary glands of HCV-infected patients are typically diffuse and predominantly consist of CD8+ T-cells, although some studies have reported a predominance of CD4+ T-cells, but to a lesser extent than in Sjögren’s syndrome (SS) [52]. 

HCV-infected individuals present a higher prevalence of liver involvement and cryoglobulinemia compared to SS patients. Patients with HCV-related salivary gland dysfunction usually lack primary SS antibodies, such as anti-SSA and anti-SSB, showing, on the other side, high levels of other autoantibodies like ANA, ACA, dsDNA, and RF [52]. In a very preliminary study, Aceti et al. have explored the relationship between HCV and Sjögren’s syndrome (SS), searching for a potential overlap in clinical features between HCV-related salivary gland dysfunction and SS, concluding that HCV may have no role in the autoimmune organ damage responsible for Sjogren’s syndrome [53]. On the other side, another early study by Arrieta et al. reinforced the hypothesis that HCV infects and replicates in the epithelial cells of salivary glands of patients with Sjogren’s syndrome or chronic sialadenitis, although the underlying pathogenic mechanisms were not clear [54]. The study conducted by Brito-Zerón et al. investigates how the hepatitis C virus (HCV) influences the immunological profile of Sjögren’s syndrome (SS) patients, analyzing 783 cases. The findings reveal that SS patients with HCV exhibit distinct immunological characteristics. In fact, the prevalence of HCV infection in patients with Sjögren’s syndrome (SS) varies widely based on classification criteria. HCV-driven autoimmune response in SS is marked by a high prevalence of mixed cryoglobulins, positive rheumatoid factor (RF), monoclonal gammopathy, and low C4 levels. Significant differences in serum monoclonal expression were noted, with SS-HCV patients showing a threefold higher prevalence of circulating monoclonal immunoglobulins (mIgs), predominantly mIgMκ, linked to mixed cryoglobulinemia. SS-HCV patients also exhibited a more restrictive monoclonal expression compared to the diverse profiles in SS patients without HCV, indicating HCV’s role in clonal B-cell selection [55].

**Figure 2:**
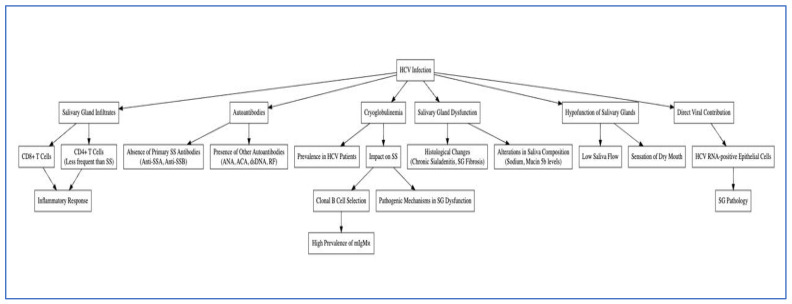
Aetiopathogenic hypotheses of the relationship between HCV infection and Xerostomia and Sjögren’s syndrome-like manifestations.

Several authors have already highlighted the absence of HCV infection in primary SS [56]. For this reason, according to the 2016 American-European Consensus Criteria, evidence of HCV infection is an exclusion criterion for the classification of a patient as having primary SS [57]. A recent study by Maldonado et al. highlighted that HCV-infected patients with xerostomia demonstrated diffuse lymphocytic infiltrates in their salivary glands, predominantly composed of CD8+ T-cells. These infiltrates were associated with significant increases in the number of inflammatory cells, suggesting an ongoing inflammatory response. The study observed chronic sialadenitis and salivary gland (SG) fibrosis in HCV-infected patients, indicative of sustained tissue damage and remodeling in response to chronic inflammation. Analysis of saliva composition revealed significant changes in sodium and mucin 5b levels. Saliva alterations suggest that HCV infection impacts salivary gland function and contributes to the sensation of dry mouth. Submandibular glands in HCV patients showed significant ultrasonographic abnormalities relative to the parotid glands, further supporting the presence of glandular pathology. The research indicated that all HCV patients examined exhibited low saliva flow, pointing to SG hypofunction, which explained the xerostomia symptoms. No significant correlation was found between the degree of lymphocytic infiltrates and the duration of HCV chronic infection. However, there was a positive correlation observed between HCV RNA-positive epithelial cells and the years of HCV infection, highlighting a direct viral contribution to SG pathology. Moreover, patients with HCV showed changes in markers of SG acinar and ductal function, consistent with the observed low saliva flow and xerostomia. The study concluded that HCV infection can cause xerostomia through mechanisms distinct from Sjögren’s syndrome. This distinct pathophysiology, reported in Figure 2, has implications for the diagnosis and treatment of xerostomia in HCV-infected individuals. Other viruses, such as hepatitis D virus, HIV-1, human T-cell leukemia virus type 1, and SARS-CoV-2, have also been associated with SG pathology and have provided comparative insights into the viral mechanisms affecting the SGs [52].

### 2.3. Periodontal Disease

Some studies have reported that periodontal conditions are exacerbated by HCV infection by an alteration of the immune response, leading to a more severe progression of gum disease [58,59]. The virus exhibits lymphotropism, being traceable in fluids like saliva (though transmission through saliva is debated due to inconsistent detection of viral RNA). The presence of HCV in saliva might be influenced by the periodontal status, with higher AST levels observed in patients with chronic periodontal disease [60]. Furthermore, HCV antigens and antibodies were studied in gingival crevicular fluid (GCF), with viral RNA and anti-HCV antibodies detected in the GCF of infected patients, suggesting a role as a source of HCV contamination in saliva [61]. Periodontal inflammation increases GCF flow and bleeding, facilitating viral migration from blood to GCF and saliva. Studies have found a high prevalence of HCV RNA in GCF, often higher than in saliva [62]. The presence of HCV in GCF probably involves infected leukocytes, necessitating further research into the molecular and cellular characteristics of GCF in HCV patients [63].

Malone et al. found that periodontal disease increased the risk of developing Alzheimer’s disease and related dementias (ADRD) among HCV patients, suggesting a link between oral health and neurodegenerative diseases in the context of HCV infection. The presence of periodontal disease was associated with a higher incidence rate of ADRD and an earlier development of these conditions in HCV patients compared to those without periodontal disease [64]. Nagao and Tsuji investigated the impact of HCV eradication on oral lichen planus (OLP) and the load of periodontal pathogens. Although it can be considered a pilot study, with only four cases presented, they found that the eradication of HCV not only improved OLP lesions but also reduced the number of periodontal pathogens, emphasizing the potential systemic benefits of HCV treatment on oral health [59]. Azatyan et al. explored clinical and morphological lesions of the oral mucosa and periodontium in viral hepatitis C, underlining the significant changes in the dental and periodontal status of patients with HCV. This study directly addresses the oral manifestations in HCV patients, providing clinical insights into the impact of HCV on oral health [65]. 

**Figure 3:**
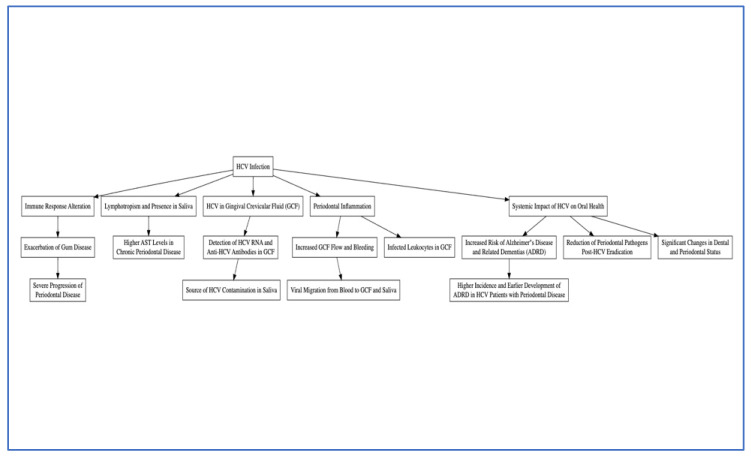
Aetiopathogenic hypotheses of the relationship between HCV infection and periodontitis.

Some other studies have expanded our understanding of the etiopathogenetic mechanisms linking periodontal diseases with viral liver diseases, particularly focusing on the role of microbiome and inflammatory processes. Chandran et al. provided a comprehensive review of the role of various viruses in periodontal disease. The review categorizes the impact of viral infections on the etiopathogenesis of periodontal disease, emphasizing the need for a better understanding of viral contributions to disease progression [66]. Gheorghe et al. (2022) discussed the dental and periodontal status of patients with hepatitis B/D. They suggested that maintaining good oral and periodontal health can limit the pathological effects of these liver diseases. They also proposed that a similar interplay could exist in HCV-related liver diseases [67]. All pathogenetic mechanisms are shown in Figure 3.

### 2.4. Head and Neck Squamous Cell Carcinoma

Recent studies have investigated the relationship between OSCC and HCV infection, providing insights into the molecular mechanisms involved and potential impacts on patient survival. Marconi et al. focused on the role of c-Myc in OSCC, indicating its significance in tumor prognosis and stem cell renewal. While it did not directly connect to HCV, the exploration of molecular pathways in OSCC offers insights into potential areas where HCV-related mechanisms might intersect, especially considering the role of c-Myc in various cancers, including those potentially influenced by viral infections [68]. Direct prevalence numbers linking OSCC specifically with HCV infection are less commonly reported and vary significantly based on population studies and the specific criteria used for diagnosing OSCC. However, studies proposed HCV infection as a risk factor for the development of OSCC, potentially due to its role in promoting chronic inflammation and its effects on cellular pathways related to cancer development [69]. In the study of Nagao et al. involving 60 patients, 35% developed multiple primary cancers (MPCs), with a notably higher incidence (62.5%) among those with HCV infection compared to those without (25%). The analysis revealed HCV as a significant risk factor for MPCs alongside primary OSCC, HCC being prevalent among HCV-positive cases. Age over 70, staging IV, and HCV positivity were identified as significant risk factors. The findings underscore the importance of comprehensive medical treatment for HCV-infected OSCC patients in Japan to mitigate the risk of developing HCC and suggest the necessity of monitoring for MPCs beyond the liver, especially given the observed hyperinsulinemia in HCV-positive patients [7]. A study by Fu-Hsiung Su et al. [70] highlighted a significant association between HCV infection and an increased risk of oral cavity cancer. Conducted within a Taiwanese population, this nationwide cohort analysis reveals that individuals with HCV are at a notably higher risk of developing oral cavity cancer compared to those without viral hepatitis. This association is particularly pronounced among adults aged 40–49, underscoring the importance of vigilant oral health monitoring in HCV-infected patients to potentially mitigate cancer risk. The incidence of oral cavity cancers was 2.28-fold higher among patients with HCV alone than non-viral hepatitis group (6.15 versus 2.69 per 10,000 person-years). After adjusting for sociodemographic covariates, HCV alone was significantly associated with an increased risk for oral cancer. However, the study does not specify whether the study population also suffered from liver cirrhosis. This aspect is not negligible when conducting a study of this type and therefore limits the results. In a 2004 study on an Italian cohort of 402 patients with OLP, the relative risk of OSCC for patients with HCV as compared with those without HCV infection was 3.16 (0.8–12.5) [69]. However, although four out of nine patients with OSCC were HCV-infected, the increased risk was not significant, possibly because of low statistical power.

**Figure 4:**
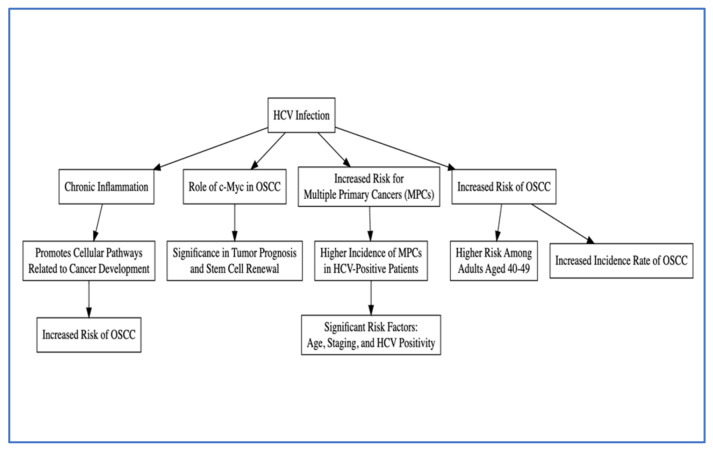
Aetiopathogenic hypotheses of the relationship between HCV infection and HNSCC.

A meta-analysis by Borsetto et al. synthesized evidence on HCV’s link to head and neck squamous cell carcinoma (HNSCC), including cancers of the oral cavity, oropharynx, hypopharynx, and larynx. Eight studies were analyzed, showing significant risk associations for oral cavity (RR = 2.13), oropharynx (RR = 1.81), and larynx cancers (RR = 2.57). Hypopharyngeal cancer also showed a trend towards risk elevation (RR = 2.15), though it was not statistically significant. The study underscores the importance of monitoring HCV-infected patients for early HNSCC detection and raises awareness of potential undiagnosed HCV in HNSCC patients [71]. All pathogenetic mechanisms are shown in Figure 4.

## 3. Materials and Methods

### 3.1. Search Strategy

An exploratory literature search was conducted using major scientific databases, including PubMed, Scopus, Web of Science, and Google Scholar, to identify relevant publications on the oral manifestations of HCV infection. The search covered literature published up to April 2023. Search terms used included “hepatitis C virus”, “HCV”, “oral manifestations”, “oral health”, “oral lichen planus”, “xerostomia”, and “periodontal disease”, with various combinations of these terms. Boolean operators (AND, OR) were employed to broaden the search.

### 3.2. Selection Criteria 

Given the narrative review’s aim to provide a comprehensive overview, studies were selected based on their relevance to the topic of oral manifestations associated with HCV infection. This included original research articles, review papers, case reports, and clinical trials. There were no strict inclusion or exclusion criteria based on study design or language to ensure a broad and inclusive selection of the literature. However, preference was given to studies that significantly contributed to understanding the relationship between HCV infection and oral health outcomes.

### 3.3. Data Collection

The data collection process involved summarizing key findings from the selected studies, focusing on types of oral manifestations reported, the prevalence among HCV-infected individuals, diagnostic approaches, and treatment outcomes. This process was more qualitative, aiming to capture the breadth of knowledge rather than quantitatively synthesizing data.

### 3.4. Analysis Method

A narrative synthesis was employed to organize and present the findings. This approach allowed for a flexible interpretation of the diverse body of literature, highlighting themes and patterns regarding oral manifestations of HCV infection, and discussing diagnostic challenges and treatment strategies.

The main results are summarized in diagrams created using RStudio (version 2024.04.2+764).

### 3.5. Ethical Considerations

As this review is based on the analysis of previously published data, no specific ethical approval was required. Nevertheless, the review was conducted with a commitment to ethical integrity, ensuring respect for the original sources and their contributions to the field.

## 4. Conclusions

The comprehensive review elucidates the potential intricate association between HCV infection and its diverse oral manifestations, notably oral lichen planus, xerostomia, and periodontal disease, underscoring the systemic impact of HCV beyond hepatic involvement. Given the present state of the scientific literature, clinical recommendations of dental practice for HCV patients have to be wide and unspecific, as there is no clear evidence for a specific type of conduct. Such findings encourage the need for heightened vigilance and regular oral health assessments among patients with chronic HCV infections, facilitating early diagnosis and timely intervention. Further studies are yet needed to reach complete scientific evidence. Future research directions should aim at unraveling the underlying pathophysiological mechanisms linking HCV to oral health conditions and refining treatment protocols to encompass comprehensive care strategies that address both hepatic and extrahepatic manifestations of HCV, ultimately enhancing patient outcomes and quality of life.
